# Mitochondrial Dysfunction in Cancer

**DOI:** 10.3389/fonc.2013.00292

**Published:** 2013-12-02

**Authors:** Michelle L. Boland, Aparajita H. Chourasia, Kay F. Macleod

**Affiliations:** ^1^The Ben May Department for Cancer Research, The University of Chicago, Chicago, IL, USA; ^2^Committee on Molecular Metabolism and Nutrition, The University of Chicago, Chicago, IL, USA; ^3^Committee on Cancer Biology, The University of Chicago, Chicago, IL, USA

**Keywords:** mitochondria, oxidative phosphorylation, mitophagy, mitochondrial biogenesis, mitochondrial fusion and fission, cancer metabolism, cell cycle control

## Abstract

A mechanistic understanding of how mitochondrial dysfunction contributes to cell growth and tumorigenesis is emerging beyond Warburg as an area of research that is under-explored in terms of its significance for clinical management of cancer. Work discussed in this review focuses less on the Warburg effect and more on mitochondria and how dysfunctional mitochondria modulate cell cycle, gene expression, metabolism, cell viability, and other established aspects of cell growth and stress responses. There is increasing evidence that key oncogenes and tumor suppressors modulate mitochondrial dynamics through important signaling pathways and that mitochondrial mass and function vary between tumors and individuals but the significance of these events for cancer are not fully appreciated. We explore the interplay between key molecules involved in mitochondrial fission and fusion and in apoptosis, as well as in mitophagy, biogenesis, and spatial dynamics of mitochondria and consider how these distinct mechanisms are coordinated in response to physiological stresses such as hypoxia and nutrient deprivation. Importantly, we examine how deregulation of these processes in cancer has knock on effects for cell proliferation and growth. We define major forms of mitochondrial dysfunction and address the extent to which the functional consequences of such dysfunction can be determined and exploited for cancer diagnosis and treatment.

## Introduction

Mitochondrial integrity is central to efficient cellular energy production and cell survival in the face of environmental stresses, such as nutrient deprivation and ischemia, but also in response to genotoxic agents, such as those used in cancer therapies (1–4). How changes in mitochondrial mass and function affects the basic biology of cancer or determine the clinical outcome for cancer patients stands out as having unexplored diagnostic and therapeutic potential.

### Mitochondria and the warburg effect

Defective mitochondria was proposed by Otto Warburg to explain his observation that tumor cells undergo increased aerobic glycolysis (the so-called “Warburg effect”) compared to normal cells (5). While mutations in key Krebs cycle enzymes support the notion that mitochondrial metabolism is inherently defective in at least a few human cancers (6), evidence that dysfunctional mitochondria are the major cause of the Warburg effect is limited (5). Instead, accumulating evidence supports altered expression and activity of key glycolytic enzymes in tumor cells. For example, altered expression of phosphoglycerate dehydrogenase, phosphoglycerate mutase 1, and pyruvate kinase M2 has been shown to reduce the rate of glycolytic flux to pyruvate and increase flux to biosynthetic pathways (7), such as serine biosynthesis (8, 9) and the pentose phosphate pathway (10). Interestingly, increased glucose metabolism and the Warburg effect also promote tumor cell survival through redox regulation of cytochrome *c* and inhibition of apoptosis (11). This may explain the Warburg effect and tumor growth without necessarily invoking defective mitochondria, as discussed elegantly in recent reviews (5, 12–18).

### Mitochondrial genome mutations in cancer

While mitochondrial dysfunction does not necessarily explain the Warburg effect, there is significant evidence that tumors do indeed accumulate defective mitochondria (19–21). Homoplasmic mutations in the mitochondrial genome have been found in primary tumors (22) and linked to both increased primary tumor growth (23) and metastasis (24). The tumor-promoting effects of mitochondrial genome mutation, such as in the genes encoding subunit 1 of cytochrome oxidase (CO) or various subunits of NADH dehydrogenase (ND) is due in part to increased levels of cytosolic and mitochondrial reactive oxygen species (ROS) resulting from electron escape from the respiratory chain when these genes products have reduced function (19–21, 23, 24). There is also evidence that more efficient electron chain activity and complex I activity in particular limits breast tumor growth and metastasis in part by maintaining high NAD+/NADH levels (25). However, as recently discussed (26), more research is required to establish the extent to which mitochondrial genome mutations actually drive tumor growth and progression, as opposed to being a marker or readout of mitochondrial dysfunction itself.

### Mitochondrial ROS in cancer

Increased ROS levels primarily emanating from the mitochondria are a noted feature of transformed cells that are variously attributed to inefficiencies in electron transport at the respiratory chain, increased metabolic demand, reduced ROS scavenging, oncogene-induced replicative stress, and altered mitochondrial dynamics (27–30). Oncogene-induced ROS promotes tumorigenesis in numerous ways, including stabilization of hypoxia-inducible factor (HIF)-α, induction of oxidative base damage to DNA, increased calcium flux, inactivation of key phosphatases, such as Pten and activation of both the NRF2 and NF-κB transcription factors (27, 29). Elevated ROS levels in tumor cells compared to normal cells has been exploited experimentally to kill cancer cells specifically by chemically pushing ROS levels over a critical homeostatic threshold that is incompatible with either growth or survival of tumor cells but tolerable by normal cells (31–33). Indeed, many current genotoxic agents used in the clinic, such as cisplatin and certain alkaloids, rely on ROS production for their efficacy (29, 34, 35). However, one major side-effect of increasing ROS systemically in a clinical setting is the damaging effect of elevated ROS on normal tissues that can lead to disrupted differentiation and defective immune cell function, particularly in cell types, such as macrophages and neutrophils, where ROS is inherently elevated to perform critical signaling roles (29). Also, not all tumor cell types are equally sensitive to ROS induction with differences in the sensitivity of epithelial cells (resistant) compared to cells of mesenchymal origin (sensitive) (32). While appreciating the important signaling role mitochondrial ROS plays in cancer etiology and treatment response, this topic has recently been thoroughly reviewed elsewhere (29) and is not the focus of this review.

### Mitochondria are highly dynamic

**Figure 1:**
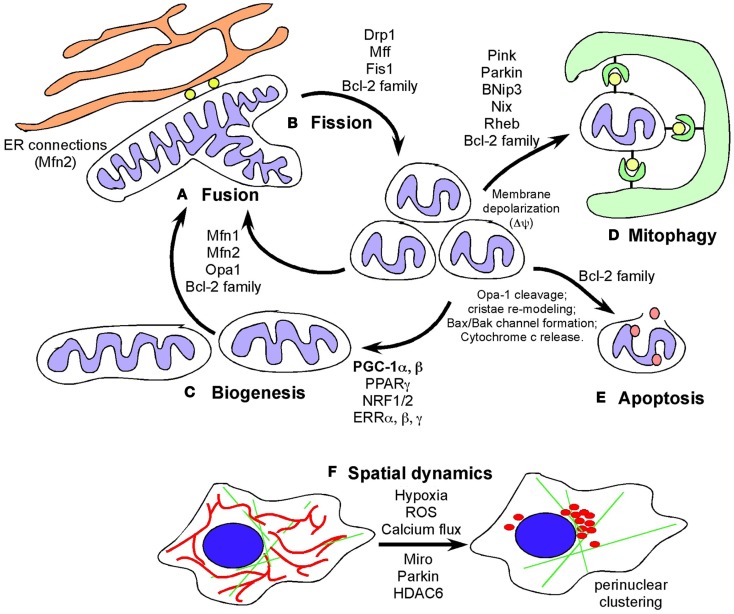
**Defining mitochondrial dynamics**. **(A)** Mitochondrial fusion requires the action of fusion proteins, OPA-1 at the IMM and Mitofusin 1 and Mitofusin 2 at the OMM promoting the fusion of membranes of juxtaposed mitochondria. Mitochondrial fusion is selective for polarized mitochondria and promoted by growth on oxidative carbon sources, such as galactose, that also induces respiratory chain protein expression, increased cristae density and formation of respiratory chain supercomplexes and increased OXPHOS. Fusion likely also contributes to increased respiration and mitochondrial metabolism by promoting increased diffusion of intermediate metabolites and reducing agents. Fusion also limits mitophagy and apoptosis. **(B)** Mitochondrial fission is promoted by the GTPase activity of the dynamin-related protein (DRP1) that is recruited to mitochondria in response to stresses, such as hypoxia, where DRP1 interacts with its mitochondrial receptors (Mff1, Fis1 and others) to pinch off mitochondria into smaller units. Mitochondrial fission depolarizes mitochondria but mitochondria generally recover. Failure to restore membrane potential is thought to target mitochondria for degradation by autophagy or depending on other stresses, result in apoptosis. Cleavage of OPA-1 promotes apoptosis. **(C)** Biogenesis is induced by nutrient deprivation and in response to oxidative stress and requires the coordinated expression of nuclear and mitochondrial encoded genes that are co-regulated by transcription factors NRF1/2, PPARγ, ERRα, β, γ, and the key transcriptional co-factor, PGC-1α. Mitochondrial biogenesis is required for cell growth to produce increased metabolites and energy and defects in biogenesis are frequently lethal to cells and organisms. **(D)** Mitophagy is a specialized form of autophagy in which mitochondria are targeted to nascent phagophores and engulfed by autophagosomes that fuse with lysosomes to degrade the encapsulated mitochondria. Mitochondrial fragmentation is required for mitophagy and induction of fusion protects mitochondria from degradation under starvation conditions. Mitophagy is promoted by a number of different mechanisms including Pink1/Parkin-mediated pathways and also the BNIP3/NIX pathway. **(E)** Apoptosis is a terminal event that is promoted by the activity of BH3-only members of the Bcl-2 superfamily of cell death regulators. When apoptosis is inhibited, novel functions for BAK/BAX and other Bcl-2 family members has been revealed. **(F)** Mitochondrial spatial dynamics is relatively under-studied but mitochondria respond to key stresses, including hypoxia and calcium flux in the cell, by changing their sub-cellular localization, including coalescing around the nucleus and changing their proximity to the ER. Mitochondrial migration in cells is modulated by Miro, a Ca^2+^ dependent small G protein as well as by poorly understood effects of Parkin and HDAC6.

Mitochondria are highly dynamic organelles responding to cellular stress through changes in overall mass, interconnectedness, and sub-cellular localization (1–3)(Figure 1). Change in overall mitochondrial mass reflects an altered balance between mitochondrial biogenesis (increased mitochondrial genome duplication combined with increased protein mass added to mitochondria) and rates of mitophagy (degradation of mitochondria at the autophagosome) (36–38). In addition, the extent to which mitochondria are interconnected to each other as a single continuous mitochondrial reticulum is determined by the extent of mitochondrial fusion while conversely, mitochondrial fission results in fragmented mitochondria of smaller overall dimensions (2, 39, 40). Mitochondria are also dynamic in terms of where they are located in the cell with increased perinuclear localization under hypoxia compared to normoxia (41), at axonal termini in neurons (42), and movement along microtubules toward lysosomes under conditions that promote mitophagy (43). The extent to which control of mitochondrial dynamics, not only rates of fission versus fusion, but also changes in mitochondrial mass and sub-cellular spatial organization (Figure 1), is deregulated in cancer has been less frequently reviewed than Warburg (5), ROS (29), and mitochondrial genome DNA (mtDNA) mutations (26) and is the focus of this review. We shall also assess whether a rational basis exists to target key aspects of mitochondrial dynamics to treat cancer.

## Fission versus Fusion in Cancer

Re-modeling of the mitochondrial network in cells is mechanically regulated by key dynamin-related fission and fusion gene products and takes place in response to hypoxia, cell cycle cues, changing energy demands, and other cellular stresses (1, 2, 39, 40, 44–46).

### The mechanics of fission and fusion

Mitochondrial fusion is promoted by homotypic/heterotypic interactions of the Mitofusin 1 and Mitofusin 2 dynamin-related GTPases at the outer mitochondrial membrane (OMM) of adjacent mitochondria and by Opa-1, also a dynamin-related GTPase, at the inner mitochondrial membrane (IMM) (1, 39, 40, 45). Inhibition or loss of any one of these proteins impedes mitochondrial fusion leading to increased mitochondrial fragmentation and is associated with clinical neuropathy in Charcot–Marie–Tooth disease and Autosomal Dominant Optic Atrophy, highlighting the critical role played by mitochondrial fusion in cellular homeostasis, particularly in the nervous system (39, 42).

Mitochondrial fission requires the recruitment of a different dynamin-related GTPase, Drp1 to the OMM where it forms ring-like oligomers that pinch off mitochondria into smaller fragmented mitochondria (39). Fission is important ahead of mitosis to ensure even distribution of mitochondria to daughter cells (47–49) but also occurs when cells undergo mitophagy or apoptosis (2, 40, 44, 50, 51). Recruitment of cytosolic Drp1 to the mitochondria during fission is a regulated process involving post-translational modification of Drp1 (49, 52–54) and its interaction with putative receptors at the OMM such as Mitochondrial Fission Factor (Mff), Fis1, MiD49, MiD51, and possibly other proteins with which Drp1 interacts (45, 55–59). Recent work has also highlighted a role for the endoplasmic reticulum (ER) that is intimately associated with mitochondria, in determining the sites at which fission will occur (60). The constriction of mitochondria at points of contact with the ER are set up prior to recruitment of Drp1 to mitochondria and independent of Mff. Intriguingly, the mitochondrial fusion protein Mfn2 also plays a role in tethering mitochondria to the ER, in a manner required for proper calcium uptake by the mitochondria from the ER (61). Screens in yeast have identified additional putative molecular regulators of mitochondrial tethering to the cell cortex and the ER in ways that regulate mitochondrial positioning in cells and inheritance by daughter cells (62) but whether similar mechanisms operate in mammalian cells is unclear.

### Stress-induced changes in rates of fission and fusion

Beyond the actual mechanics of mitochondrial fission and fusion, the signaling pathways that regulate these processes are only just coming to the fore as mechanisms that may be disrupted in cancer. These signaling pathways respond to specific stresses and serve to coordinate mitochondrial dynamics with other aspects of cellular physiology. Drugs that inhibit protein synthesis, including mTOR inhibitors, as well as other stresses such as ultra-violet (UV) light have been shown to promote so-called “stress-induced mitochondrial hyperfusion” that relies upon canonical fusion proteins (Opa-1, Mfn1) (46), although, how these stress signaling pathways activate the fusion machinery is not clear. Stress-induced hyperfusion of this kind promotes ATP production through more efficient oxidative phosphorylation (OXPHOS) (63), and also inhibits mitophagy and prevents apoptosis (46, 64, 65).

The functional consequences of altered rates of fission or fusion for cellular metabolism, cell cycle kinetics and cell viability are a “work in progress” relying on numerous systems and technical approaches. In *Drosophila*, Yorkie-mediated up-regulation of Opa-1 and mitochondrial fusion was required for Yorkie/YAP-dependent proliferation and tumorigenesis (66). In mammalian systems, glucose deprivation and use of galactose as an alternative source of carbon, resulted in cells switching from glycolysis to OXPHOS, as expected, but significantly caused a marked increase in mitochondrial fusion and an accompanying increase in respiratory chain protein expression and cristae density, without any increase in mitochondrial mass (67).

Consistent with a critical role for mitochondrial fusion in regulating metabolism, inactivation of Mitofusin 1, Mitofusin 2, or Opa-1 inhibits oxidative metabolism and cell growth (68, 69). If fusion promotes mitochondrial ATP production (63), it is reasonable to postulate that this is achieved by increasing the efficiency of the electron transport chain (ETC) that is key to OXPHOS. This may be achieved by increasing the numbers/density of ETC complexes through increased expression of key components, as has been reported (67), or by altering the composition of specific respiratory chain components into different supercomplexes to maximize utilization of specific substrates, such as NADH in the presence of glucose versus FAD in the presence of fatty acids (70). Recent data has shown that assembly of respiratory chain complexes (RCC) into supercomplexes and increased respiratory efficiency is promoted by tighter cristae formation that is dependent on the fusion protein, Opa-1 (71). Conversely, disruption of cristae formation by knockout of Opa-1 in mice promoted mitochondrial fragmentation, cristae dissolution, and reduced RCC formation and respiration (71). Thus fusion may promote respiration through Opa-1 dependent effects on cristae density and formation of respiratory chain supercomplexes. Additionally, fusion may induce mitochondrial membrane potential changes that promote uptake of pyruvate or other substrates that fuel OXPHOS. Clearly, a more continuous mitochondrial lumen achieved by increased fusion is likely to promote more rapid diffusion of ADP, NADH, FADH_2_, and other matrix metabolites required to drive more efficient OXPHOS. In this way, fusion would also likely promote increased carbon flux through the Krebs’ cycle, more efficient rates of fatty acid oxidation and possibly increased activity of other metabolic pathways located at the mitochondria.

It has been suggested that fusion may promote respiratory efficiency by promoting complementation of mtDNA mutations (51). While this may be the case in some instances, the failure to detect homoplasmic mutations in mtDNA encoded subunits of CO, ND, ATP synthase, or cytochrome *b* in primary cells, or indeed more widely in tumor cells suggests that complementation of mtDNA mutations is not the key role of mitochondrial fusion in metabolism. Indeed, increased fusion has been shown to promote OXPHOS in very short time frames (67), indicating that the effects of fusion on oxidative metabolism are post-translational and not primarily dependent on gene complementation between mitochondrial genomes.

The effects of mitochondrial fusion in promoting oxidative metabolism at the mitochondria imply a contrasting role for mitochondrial fission in inhibiting oxidative metabolism, perhaps by decreasing substrate uptake, disrupting cristae, and respiratory complex formation and/or limiting diffusion of reducing equivalents. Oxygen is the major electron acceptor from complex IV of the respiratory chain, and thus it benefits the cell to decrease OXPHOS under limiting oxygen conditions (hypoxia) both to maximize efficient use of the smaller amounts of oxygen available but also to prevent generation of damaging ROS. Hypoxia limits OXPHOS in a number of ways but promoting mitochondrial fission may be one of the key mechanisms. Hypoxia promotes mitochondrial fission by modulating Drp1 activity and interaction with Fis1 (72). Hypoxia-induced expression of Siah2, an E3 ubiquitin ligase (73) targets the mitochondrial scaffold protein, anchoring protein 121 (AKAP121) for degradation (72) thereby preventing protein kinase A (PKA) dependent inhibition of Drp1. These observations, amongst others, are consistent with an inhibitory role for mitochondrial fission in OXPHOS. Increased fission linked to deregulated expression of Drp1 (increased) and Mfn2 (decreased) has been observed in tumor cells (74) but to what extent this contributes to the Warburg effect or other aspects of tumor growth remains to be determined.

### Coordination of rates of fusion/fission with cell cycle

Several reports indicate that increased mitochondrial fusion is required not only for efficient oxidative metabolism (68, 69) but is necessary for proliferation and entry into S-phase (63). In this latter study, mitochondrial membrane polarization and hyperfusion of mitochondria occurring at the G1/S transition was required for cyclin E (CCNE) expression and S-phase entry (63). Additional studies have also shown that mitochondrial bioenergetics are linked to cell cycle progression (75). However, artificially inducing hyperfusion, either through treatment with mdivi (a drug that inhibits Drp1) (63) or expression of dominant negative Drp1 (76), resulted in untimely induction of hyperfusion and sustained cyclin E over-expression at inappropriate phases of cell cycle, such as G2/M (76). This in turn was accompanied by replication stress, DNA damage, centrosomal amplification, and chromosomal instability (63, 76), all known features of cells over-expressing cyclin E (77). Interestingly, while cyclin E over-expression was required for proliferation and genome instability arising from hyperfusion or dysfunctional fission (63, 76), the specific factors produced by hyperfusion that resulted in cyclin E up-regulation have not been identified. Increased ROS has been previously reported to modulate levels of key cell cycle proteins, such as Emi-1 (78) but neither increased mitochondrial ROS nor altered ATP production (76) explain increased proliferation induced by mitochondrial hyperfusion (although this arguably requires further validation), leaving us with the unanswered question of what drives cyclin E expression in these circumstances.

Glycolysis is also cell cycle regulated and increases at the G1/S-phase boundary due in part to stabilization of 6-phosphofructo-2-kinase/fructose-2,6-bisphosphatase, isoform 3 (Pfkfb3) that is normally turned over by APC/Cdh1 in early stages of G1 phase of cell cycle (79). Interestingly, Drp1 is also turned over by APC/Cdh1 in G1 phase of cell cycle in a CDK-dependent manner (47, 49) suggesting a possible mechanism by which up-regulation of glycolysis may be coordinated with mitochondrial dynamics. Evidence that glutaminolysis is cell cycle regulated is sparse but since c-Myc and RB/E2F both modulate glutamine uptake (80–83), it would not be surprising if glutaminolysis, like glycolysis, was up-regulated as cells enter S-phase.

### Dual role of Bcl-2 family members in mitochondrial dynamics and apoptosis

**Figure 2:**
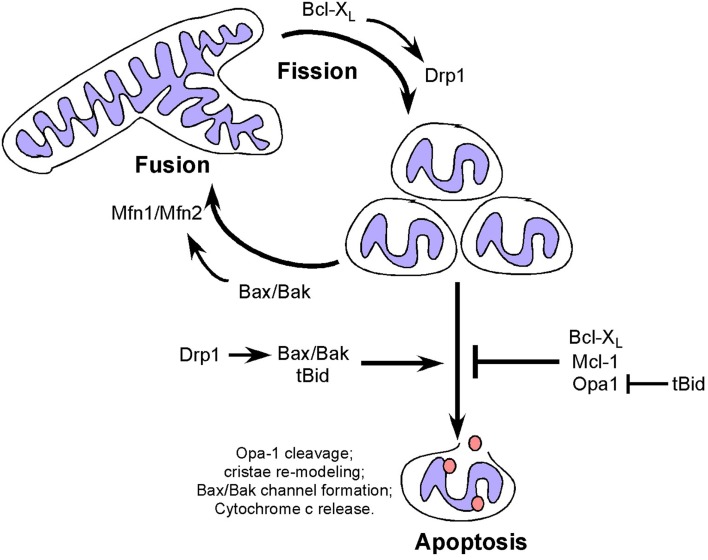
**Dual and apparently opposing roles of Bcl-2 family members and fission/fusion proteins in apoptosis and mitochondrial dynamics**. Mitochondrial fission and fusion proteins appear to modulate apoptosis through activities that are distinct from their roles in mitochondrial dynamics but which involve members of the Bcl-2 family. Conversely, Bcl-2 family members modulate mitochondrial fission and fusion in a manner that appears to be independent of their functions in apoptosis.

In addition to effects on cellular metabolism and cell cycle, mitochondrial fusion can delay cytochrome *c* release and apoptosis (46, 84), with Opa-1 oligomerization inhibiting pro-apoptotic cristae remodeling independent of its role in fusion (85) (Figure 2). Disruption of Opa-1 oligomers in a Bax/Bak-dependent manner by the pro-apoptotic BID protein was independent of mitochondrial membrane permeabilization (86) but was associated with pro-apoptotic cristae remodeling (a requirement for cytochrome *c* release) and Opa-1 deficient cells are more susceptible to apoptosis (87, 88). Interestingly, prohibitins promote cellular proliferation and resistance to apoptosis by inhibiting OPA-1 cleavage at the IMM thereby promoting fusion and normal cristae morphology (89).

In contrast to mitochondrial fusion, mitochondrial fission promotes mitochondrial membrane depolarization, cytochrome *c* release and apoptosis (90), with Drp1 promoting Bax oligomerization through mechanisms independent of its GTPase activity (91), possibly explaining how fragmented mitochondria are more amenable to Bax/Bak channel formation. Thus both mitochondrial fission and fusion proteins appear to modulate apoptosis through activities that are distinct from their roles in mitochondrial dynamics but which involve members of the Bcl-2 family (Figure 2).

Members of the Bcl-2 superfamily of cell death regulators are extensively characterized for their key role in regulating apoptosis (2, 44) but they also have an emerging role in mitochondrial dynamics (44, 92, 93). Bak and Bax are essential for apoptosis, such that Bax/Bak double knockout cells are resistant to apoptosis (94, 95). When Bak and Bax are activated by pro-apoptotic signals, they undergo oligomerization to form a channel in the OMM through which cytochrome *c* is released resulting in the formation of the apoptosome and activation of caspases (96). Apoptosis-resistant Bax/Bak null cells exhibit extensive mitochondrial fragmentation that is rescued by over-expression of Bak in the absence of apoptotic signaling suggesting that Bak and Bax can promote mitochondrial fusion (92). Indeed, the soluble form of Bax interacts directly with Mfn2 to promote its GTPase activity while both Bak and Bax interact with Mfn1 (90, 92, 97). Conversely, the anti-apoptotic Bcl-X_L_ has been shown to promote mitochondrial fission in neurons through interactions with Drp1 that promote its GTPase activity (98). More recently, Mcl-1 has been implicated in modulating mitochondrial dynamics through an amino-terminal truncated form that localizes to the mitochondrial matrix, in contrast to full-length Mcl-1 at the OMM (99). Truncated Mcl-1 in the matrix is required for mitochondrial fusion and assembly of the F_0_F_1_-ATP synthase and for efficient respiration (99). This novel function for Mcl-1, distinct from its anti-apoptotic function may explain the observed heart failure in Mcl-1 null mice, in which cardiomyocytes exhibited aberrant mitochondrial structure and defects in respiration that were not rescued by Bax/Bak deletion (100), although the defects were partially rescued by deletion of cyclophilin D, a key regulator of the mitochondrial permeability transition pore (101). Thus there is regulation of the apoptotic activity of Bcl-2 related proteins by fusion/fission proteins and conversely regulation of fission/fusion protein activity by Bcl-2 related proteins. What is not clear is whether the activities of Bcl-2 proteins, and Bax/Bak in particular, in apoptosis and fission/fusion are exclusive. The increase in mitochondrial fragmentation taking place during apoptosis suggests that the pro-fusion activity of Bak/Bax is suppressed by their pro-apoptotic functions but formal experimental evidence supporting this is lacking. Similarly, it is not known whether reduced OXPHOS resulting from increased fission may contribute to cellular susceptibility to apoptosis. Finally, it is not clear whether altered mitochondrial dynamics contributes to the oncogenic activity of key Bcl-2 family members, such as Bcl-2 or Bcl-X_L_ that are both over-expressed in certain human malignancies (102). For example, increased Bcl-X_L_ expression in tumors may promote mitochondrial fission, as seen in neurons (98), that in turn would be predicted to limit mitochondrial OXPHOS thereby promoting the Warburg effect.

## Altered Mitochondrial Mass in Cancer – Biogenesis versus Mitophagy

**Figure 3:**
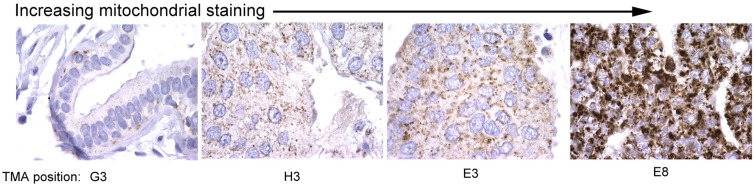
**Variation in mitochondrial staining in human breast cancers**. Immunohistochemical staining for mitochondrial 60 kDa antigen (Biogenex clone 113-1) reveals marked variations in mitochondrial staining between different primary human breast cancers with some tumors showing very low staining (left) and others very high staining (right). Differences in mitochondrial mass between different primary tumors examined in this study was greater than intra-tumor heterogeneity in mitochondrial mass. The significance of these differences in mitochondrial mass for tumor growth, progression, and therapy response is unknown.

Mitochondrial mass in cells is regulated by both changes in mitochondrial biogenesis and mitophagy, two processes that are tightly regulated in response to cellular stress, including nutrient availability, oxidative damage, and redox state. Mitochondrial biogenesis and mitophagy are both influenced by the activity of key oncogenes and tumor suppressors, and much has recently been discovered about how these processes are coordinated in cultured cells and in mouse models. However, surprisingly little evidence is available that examines changes in mitochondrial mass in primary tumors *in vivo*. Based on what is known about regulation of mitochondrial mass in tumor cell lines, one might expect that changes in cancers might be linked to specific oncogenic lesions, for example c-Myc amplification, or to localized regional effects of hypoxia/ischemia, or indeed be reflective of or contribute to evolving tumor heterogeneity. Our data identifies significant variation in mitochondrial mass between tumors in different individuals (Figure 3) but whether this relates to tumor grade, molecular sub-type, therapy response, and/or recurrence-free survival is not clear. However, this would be clinically relevant if it allowed improved stratification of cancer patients for treatment purposes.

### Mitochondrial biogenesis in cancer

Mitochondrial biogenesis involves replication of the mitochondrial genome and coordinated expression of both nuclear and mitochondrial encoded gene products (3, 103, 104). Critical nuclear-encoded mitochondrial proteins, such as Tfam, Tfb2, and the mitochondrial RNA polymerase (Polrmt) are translated in the cytosol and encode a mitochondrial targeting sequence allowing their regulated import into the mitochondrial network where they are sorted for function (105). Key ETC proteins (subunits of ND and cytochrome *b*, for example) are encoded by the mitochondrial genome and since these proteins are translated in the mitochondrial matrix, coordinated induction of mitochondrial encoded tRNAs and rRNA expression is also required (104). The entire process is highly responsive to redox stress, nutrient availability, and mitochondrial function (104, 106) and defective mitochondrial biogenesis results in embryonic lethality and disease (3, 107). Mitochondrial mass increases in proportion to cell size (108) although opinions differ on whether mitochondrial biogenesis is cell cycle regulated (109, 110).

**Figure 4:**
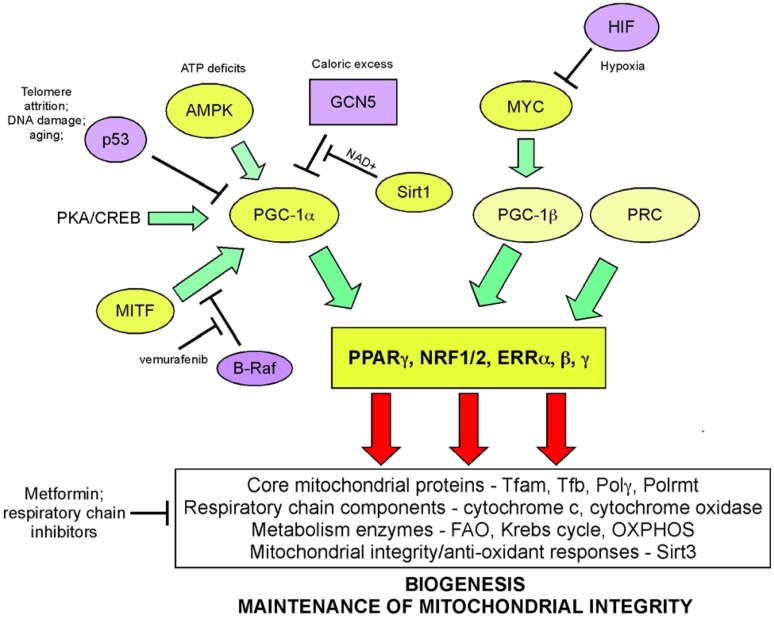
**Signaling pathways regulating biogenesis in response to stress**. Mitochondrial mass is increased in response to nutrient stress through increased mitochondrial biogenesis. The peroxisome-proliferator activator receptor-gamma co-activator 1-alpha (PGC-1α) is key to coordinating responses to nutrient availability and induction of biogenesis through its co-activation of NRF1/2, ERRα, β, γ, and PPARγ. These transcription factors induce expression of key nuclear-encoded genes, such as Tfam and mitochondrial polymerases, but also induce expression of metabolic enzymes active at the mitochondria and other proteins that are imported into the mitochondria. Mitochondrial encoded proteins required for respiratory chain function are also induced secondary to the actions of PGC-1α and its related proteins, PGC-1β, and PRC. Both p53 (through inhibition of PGC-1α) and c-MYC (through activation of PGC-1β) modulate biogenesis. Recent data highlights a role for MITF-induced PGC-1α activity and mitochondrial metabolism in a subset of human melanomas, that is sensitive to B-Raf inhibitors (since B-Raf blocks the action of MITF on PGC-1α).

Mitochondrial biogenesis depends upon the activity of a hierarchy of nuclear transcription factors, that includes peroxisome-proliferator activator receptor-alpha (PPARα), PPAR-γ, nuclear respiratory factor 1 (NRF1), nuclear respiratory factor 2 (NRF2), and estrogen related receptors (ERR) α, β, γ (36, 103). NRF1 and NRF2 both modulate expression of respiratory chain components, such as cytochrome *c* and CO subunits in addition to anti-oxidant genes, while ERR factors regulate expression of genes involved in fatty acid oxidation, Krebs cycle, and OXPHOS (103). All of these transcription factors are critically dependent for their activity on PPAR-γ co-activator 1-alpha (PGC-1α) that was first identified as a co-activator of PPARγ in brown adipocytes. PGC-1α is now recognized as the major integrator of transcriptional responses to nutrient stress (Figure 4) that together with structurally and functionally related proteins, PGC-1β and PRC (PGC-related co-activator) promotes mitochondrial biogenesis, cellular metabolism, and anti-oxidant responses through coordinated activation of the afore-mentioned transcription factors, including NRF2, ERRα, and PPARγ (103, 106).

Nutrient supply and energy balance in the cell modulates PGC-1α activity at both the transcriptional and post-translational level (106) (Figure 4). Cells respond to mitochondrial damage by increasing mitochondrial biogenesis and this is also dependent on up-regulated PGC-1α expression (111, 112). PGC-1α is transcriptionally activated by PPARs, mTOR (acting on YY1), and CREB (downstream of PKA signaling) leading to increased mitochondrial biogenesis (106). At the post-translational level, PGC-1α is regulated by both phosphorylation and acetylation events. Phosphorylation by the energy sensor AMP-dependent kinase (AMPK) activates PGC-1α while GCN5-mediated acetylation inhibits PGC-1α activity (106). Deacetylation of PGC-1α by NAD+ dependent SIRT1 promotes mitochondrial biogenesis and ensures that the activity of PGC-1α is sensitive to both the energy and the redox balance in the cell (113). PGC-1α co-activation of ERRα in turn promotes expression of mitochondrial SIRT3 that ensures effective scavenging of ROS at the mitochondria through activation of mitochondrial superoxide dismutase, amongst other mitochondrial sirtuin targets (114).

Key tumor suppressors and oncogenes regulate mitochondrial biogenesis. The c-Myc oncogene stimulates mitochondrial biogenesis through induction of PGC-1β expression (115) leading to increased expression of key mitochondrial proteins, including Tfam, Polγ, and NRF1 (116, 117). Regulation by c-Myc may explain how certain aspects of mitochondrial biogenesis, such as mtDNA replication appears to be cell cycle regulated, at least in some systems (109). HIF-1 by contrast inhibits biogenesis by promoting c-Myc degradation and by activating Mxi-1, a repressor of c-Myc transcriptional activity (115). Together with the role of HIF-1 in promoting mitophagy (as will be discussed later), HIF-1 mediated repression of mitochondrial biogenesis explains in part how mitochondrial mass is reduced in response to hypoxia. HIF-1α protein is stabilized by loss of SIRT3, a downstream target of PGC-1α (118), suggesting that signals that promote biogenesis, such as starvation-induced PGC-1α activity act in part to block ROS-induced HIF-1 stabilization and its inhibitory effect on biogenesis.

Increased PGC-1α expression has recently been implicated in the etiology of a subset of human melanomas as a result of its induction by the melanocyte-specific transcription factor, MITF (119, 120) (Figure 4). PGC-1α expressing melanomas exhibited high expression of mitochondrial proteins and a dependence on oxidative metabolism, that contrasted with PGC-1α low-expressing melanomas that were more glycolytic (119, 120). PGC-1α expressing melanomas were highly dependent on PGC-1α for growth and progression, possibly to protect against ROS-induced apoptosis (120). Intriguingly, the key melanoma oncogene, B-Raf was shown to suppress oxidative metabolism by inhibiting MITF-induced induction of PGC-1α and melanomas treated with B-Raf inhibitors, such as vemurafenib, were critically dependent on oxidative metabolism for survival suggesting that inhibitors of mitochondrial metabolism may synergize with B-Raf inhibitors in melanoma therapy (119). An obvious choice of such a drug is Metformin/Phenformin that inhibits complex I of the respiratory chain and is already approved for the treatment of type II diabetes.

PGC-1α has also been shown to drive HIF-independent expression of VEGF through co-activation of ERR-α and thus promote angiogenesis, although the relevance of these findings for cancer has not been directly tested (121).

Recent work demonstrated that PGC-1α is transcriptionally repressed by p53 in response to telomere dysfunction (122), possibly explaining reduced mitochondrial biogenesis during the aging process and significantly, mitochondrial dysfunction and elevated ROS in cancer when p53 is mutated (123, 124). When telomerase was inactivated in Atm null T cell lymphomas, tumors initially grew more slowly but over time, more aggressive tumors emerged that had activated the alternative lengthening of telomeres (ALT) pathway (125). Intriguingly, PGC-1β showed consistent copy-number alteration in ALT+ tumors and increased expression of PGC-1β and its targets, NRF2, TFAM, SOD2, and catalase were also detected (125) suggesting that there was a selective advantage to emerging tumors of increasing both mitochondrial biogenesis and ROS scavenging. ALT+ tumors showed increased mitochondrial dysfunction and ROS, possibly as a result of transcriptional inhibition of PGC-1α by p53 earlier in the pathogenesis of these tumors (122).

In summary, evidence suggests that mitochondrial biogenesis is tumor promoting by increasing metabolite and energy generation, and indeed biogenesis is positively regulated by the c-Myc oncogene and repressed by the p53 tumor suppressor. However, a different argument suggests that the production of new healthy mitochondria may be tumor suppressive by promoting oxidative metabolism, limiting ROS and HIF-α stabilization. Thus, whether mitochondrial biogenesis promotes or limits cancer may depend on context, such as tissue type, stage of tumor progression or on specific exogenous stresses present in the microenvironment.

### Mitophagy in cancer

Macro-autophagy is a catabolic process that plays a housekeeping role in eliminating protein aggregates and malfunctioning organelles, such as mitochondria, and is also activated in response to nutrient deprivation to provide energy (126–130). The specialized form of autophagy in which mitochondria are specifically targeted for degradation at the autophagolysosome is known as *mitophagy*. Numerous studies indicate that mitochondrial fragmentation and mitochondrial membrane depolarization precede mitophagy (64, 65, 131) and it has been suggested that mitophagy and fusion are opposing fates of dysfunctional mitochondria (43). Mitochondrial fission is a major source of depolarized mitochondria and conversely fusion is selective for polarized and respiring mitochondria (131, 132). In response to nutrient deprivation, healthy mitochondria are protected from mitophagy by mitochondrial fusion resulting from PKA induced down-regulation of Drp1 activity (64, 65) while dysfunctional/depolarized mitochondria appear to be specifically targeted for degradation possibly due to selective depletion of the fusion protein Opa-1, that is proteolytically cleaved in response to depolarization (133, 134).

**Figure 5:**
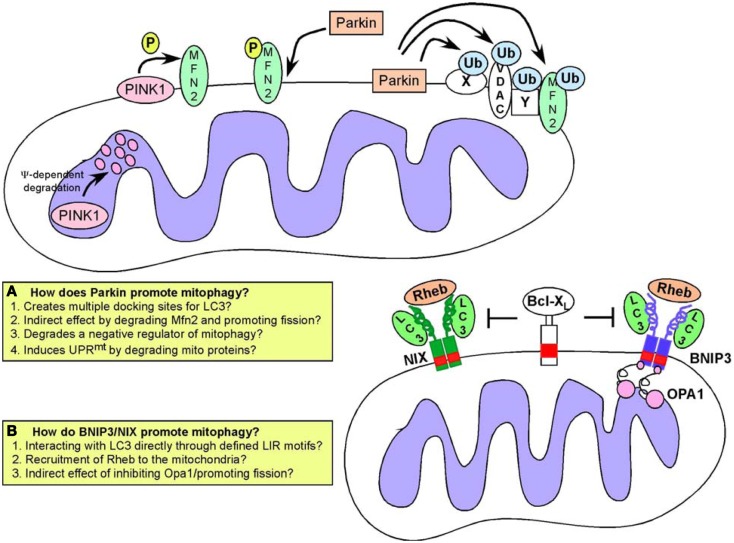
**Mitophagy pathways**. Turnover of mitochondria at the autophagosome (mitophagy) is required to maintain a healthy pool of mitochondria. Defects in mitophagy result in accumulation of dysfunctional mitochondria. Two major pathways have been identified that regulate targeting of mitochondria to the autophagosome: **(A)** the Pink1/Parkin pathway in which activation of Pink1 kinase following mitochondrial depolarization leads to phosphorylation of Mitofusin by Pink1, that then acts as a receptor for Parkin. Recruitment of Parkin, a E3 ubiquitin ligase, results in ubiquitination of multiple mitochondrial substrates but how this leads to mitophagy is still a matter for debate, and possible explanations for how Parkin functions are highlighted in the inset box; **(B)** An alternative pathway involves the activity of BNIP3 and NIX, both of which are hypoxia inducible but also regulated by FoxOs, E2Fs, and p53. BNIP3 and NIX have both been shown to interact directly with processed LC3 through a conserved LIR motif in their amino terminal ends. This interaction has been proposed to explain how BNIP3 and NIX target mitochondria to the autophagosome. Both BNIP3 and NIX are also known to interact with Rheb, and with Bcl-2/Bcl-X_L_ but the significance of these interactions for mitophagy are not clear.

Following on from fragmentation and depolarization, mitochondria are targeted to phagophore membranes through a growing number of mechanisms (Figure 5) including the Parkin/Pink1 gene products that are mutated in human Parkinson’s Disease (38) as well as the BNIP3/NIX proteins that are hypoxia-inducible and regulated by key tumor suppressors such as RB and p53 (135).

### Parkin and PINK1 in mitophagy

PINK1 (Pten-induced putative kinase-1) is a serine/threonine kinase that undergoes voltage-dependent proteolysis at the IMM in healthy mitochondria but accumulates at the OMM in response to depolarization (136, 137). PINK1 phosphorylates the fusion protein Mfn2 at the OMM and phosphorylated Mfn2 then acts as a receptor for the E3 ubiquitin ligase Parkin (138) selectively recruiting Parkin to damaged mitochondria from the cytosol (139, 140). Parkin has a number of substrates at the OMM including its own receptor, Mfn2 but also Mfn1, voltage-dependent anion channel (VDAC), and Miro (38, 141, 142). These proteins are ubiquitinated by Parkin and it was originally proposed that Parkin-mediated ubiquitination of such proteins created a docking site for the LC3-interacting protein p62/SQSTM1 (143–145) thereby linking Parkin activity to mitochondrial degradation at the autophagosome. However, it is now clear that a number of Parkin substrates are targeted for degradation by the ubiquitin-proteasome system independent of autophagy-mediated degradation (141). Furthermore, no single Parkin substrate has been shown to be required for mitophagy (146) leading to the suggestion that Parkin promotes mitophagy indirectly by either promoting fragmentation (through degradation of Mfn1/Mfn2) or by removing a negative regulator of mitophagy from the surface of the mitochondria (38, 141) (Figure 5). Alternatively, by promoting degradation of mitochondrial proteins, Parkin may be inducing an imbalance in mitochondrial versus nuclear-encoded proteins (particularly proteins involved in respiration) that has been shown to induce the mitochondrial unfolded protein response (UPR^mt^). This in turn activates adaptive mitochondrial stress signaling that is reported to improve “fitness” and promote longevity (147), so-called “mitohormesis” (123, 148, 149). Mitophagy along with mitochondrial biogenesis may be part of such compensatory mechanisms needed to restore mitochondrial function, although this has not been properly examined.

In addition to promoting mitochondrial turnover through increasing fragmentation, Parkin also modulates transport of mitochondria along microtubules to a perinuclear region where autophagosomes are concentrated (140, 146). This may be due to Parkin-mediated turnover of Miro, a protein required to tether kinesin motor protein complexes to the OMM (142). Additionally, HDAC6, a ubiquitin-binding protein deacetylase is recruited to mitochondria by Parkin activity where it promotes autophagosome-lysosome fusion and trafficking of mitochondria along microtubules (144, 150). Interestingly, a more recently discovered Parkin substrate termed PARIS represses mitochondrial biogenesis through transcriptional inhibition of PGC-1α expression (151), consistent with multiple roles for Parkin in mitochondrial homeostasis. Clearly, there is still much to be understood about the significance of these interlinking functions of Parkin/Pink1 in mitochondrial dynamics, mitophagy, and biogenesis for cellular physiology.

Human Parkin has been mapped to a common fragile site at chromosome 6q25–q26 that is found deleted in ovarian, lung, and breast cancer (152) and Parkin mutant mice are susceptible to spontaneous liver tumors (153). Parkin has also been shown to promote lipid uptake by hepatocytes, by modulating turnover of the fatty acid binding protein CD36. Further, Parkin null mice are resistant to weight gain and insulin resistance induced by feeding a high-fat diet (154). However, it is not clear whether this function in lipid metabolism in the liver relates to the function of Parkin in preventing liver tumors in mice. Parkin has also been identified as a p53 target gene and reported to prevent the Warburg effect and promote oxidative metabolism, likely through effects on mitochondrial integrity (155) providing another mechanism to explain how Parkin functions as a tumor suppressor. However, the role of Parkin as a tumor suppressor is at odds with data suggesting that mitophagy is tumor-promoting and required to maintain a healthy pool of mitochondria that are functional for TCA cycle and other aspects of metabolism upon which tumor cells depend for growth (156, 157). However, these latter studies inhibited macro-autophagy generically, not just mitophagy, and while the presence of abnormal mitochondria and defective mitochondrial metabolism suggested that defective mitophagy played a part in the observed retardation of tumor growth, the contribution of defects in turnover of ER, peroxisomes, or protein aggregates to the tumor phenotype was not examined.

### BNIP3 and NIX in mitophagy and mitochondrial integrity

The hypoxia-inducible genes BNIP3 and NIX (also known as BNIP3L) are also implicated in promoting mitophagy (135, 145, 158) (Figure 5). BNIP3 and NIX function as redox-resistant homo-dimers at the OMM and their integration into the OMM is dependent on dimerization. Both BNIP3 and NIX were originally thought to function as BH3-only proteins to promote cell death (135) but more recent work indicates that the BH3 domain in these proteins is weakly conserved and redundant for function (159). Additionally, several normal tissues express these proteins at high levels in the absence of cell death (160, 161) and it is now likely that additional signals/stresses are required for cell death associated with over-expression of either BNIP3 or NIX (162, 163). More consistent with a role on the adaptive response to hypoxia (164), both BNIP3 and NIX have been shown to promote mitophagy through interactions with LC3-related molecules and to possess an LC3-interacting region (LIR) at their unstructured amino-termini (165, 166). Thus, it has been proposed that, similar to ATG32 in yeast (167, 168), BNIP3 and NIX act as molecular adaptors targeting mitochondria directly to the autophagosome for degradation. NIX has been shown to be required for mitochondrial clearance from maturing reticulocytes (169, 170), while BNIP3 is involved in modulating mitochondrial integrity through mitophagy in skeletal muscle and liver (161, 171).

Interestingly, both BNIP3 and NIX interact with Bcl-2 and Bcl-X_L_ through their amino terminal 49 amino acids (172) suggesting that binding of BNIP3/NIX to Bcl-2 or Bcl-X_L_ may interfere with binding to processed LC3 that is dependent on an overlapping LIR motif. While reducing mitochondrial mass in cells in response to hypoxia seems likely to benefit the cell in terms of preventing excess ROS generation when oxygen is limiting (164), it is not clear whether the interaction of mitochondrial BNIP3 and/or NIX with processed LC3 at the autophagosome is a regulated interaction since not all mitochondria are turned over by mitophagy under hypoxic conditions (173). It remains to be tested whether additional events at the mitochondria, such as elevated ROS, membrane depolarization, or indeed altered electron flux at the respiratory chain modulates BNIP3/NIX structure to induce interactions with LC3 or other proteins involved in mitophagy. While not yet fully elucidated, it appears that in addition to its role in regulating mitophagy through interactions with LC3-related proteins, that BNIP3 may modulate OXPHOS and lipid metabolism in additional ways that are relevant to understanding tumor metabolism and disease (161, 174, Chourasia et al., under review).

As is observed for mitophagy involving Parkin activity, BNIP3 associated mitophagy is preceded by mitochondrial fragmentation and perinuclear clustering of mitochondria under hypoxic conditions (41, 173). Over-expression of exogenous BNIP3 promotes mitochondrial fragmentation without necessarily inducing mitophagy. This has been attributed to the inhibitory interaction of BNIP3 with Opa-1 resulting in disruption of Opa-1 complexes and cristae remodeling (175, 176). BNIP3 has also been reported to induce translocation of Drp1 to mitochondria and over-expression of either Mfn1 or dominant negative Drp1 inhibited both mitochondrial fragmentation and mitophagy induced by BNIP3 (56). This work also reported that BNIP3 induced Parkin translocation to the mitochondria in a Drp1-dependent manner. Similar to BNIP3, NIX has also been shown to promote Parkin recruitment to mitochondria (177). Parkin recruitment in this manner may reflect indirect effects of BNIP3/NIX on mitochondrial membrane potential and it remains to be determined to what extent BNIP3 or NIX depends upon Parkin to promote mitophagy.

BNIP3 has also been reported to interact with Rheb, a small GTPase that acts positively upstream of mTOR to promote cell growth (178). Similar to the interaction of BNIP3 with Bcl-2 and Bcl-X_L_ (172), Rheb was reported to interact with BNIP3 in a manner dependent on the transmembrane domain of BNIP3 consistent with Rheb only interacting with BNIP3 dimers at the OMM. Rheb binding also required the 30 amino terminal residues of BNIP3 (178) suggesting that Bcl-2 and Bcl-X_L_ may modulate the BNIP3-Rheb interaction although this has not been examined. It was suggested that BNIP3 repressed Rheb activity resulting in reduced mTOR activity and slower cell growth (178) but it is not clear if this is consistent with the interaction of BNIP3 with Rheb taking place exclusively at the OMM.

Interestingly, Rheb has recently been implicated in modulating mitophagy independent of mTOR in response to the altered metabolic state of the cell (179). Growth of cells under conditions that promote high levels of OXPHOS recruited Rheb to the OMM where it was shown to interact directly with NIX and with processed LC3. Furthermore, over-expression of Rheb in cells promoted LC3 processing and increased mitophagy (179). Intriguingly, this function of Rheb appeared to be independent of mTOR activity but dependent on NIX expression (179). This work suggested that NIX plays a role in recruiting Rheb to mitochondria under conditions of high OXPHOS leading to increased mitophagy required to maintain a healthy pool of mitochondria under high rates of oxidative metabolism. This contrasts with the previous study showing a role for BNIP3 in repressing Rheb activity (178) and while it is possible that NIX functions distinctly from BNIP3 with regards to regulation of Rheb, further work is needed to reconcile these disparate findings.

Expression of both BNIP3 and NIX is tightly regulated. Both are hypoxia-inducible HIF target genes (180, 181) although BNIP3 is more readily induced by relatively small decreases in oxygen compared to NIX that is only induced at much lower oxygen levels; an observation that is attributed to the differential dependence of BNIP3 and NIX expression on the two different transactivation domains of HIF-1α (182–184). They both also show markedly different tissue-specific patterns of expression with BNIP3 most strongly expressed in heart, liver and muscle while NIX is expressed strongly in hematopoietic tissues and in testes (160, 161). In addition to transcriptional regulation by HIFs, BNIP3 is regulated by RB/E2Fs (173), NF-κB (185), FoxO3 (171), oncogenic Ras (186, 187), and p53 (188), while NIX is also regulated by p53 (189).

Both BNIP3 and NIX are deregulated in human cancer with elevated expression of both genes detected at pre-malignant stages of several different tumor types, but they appear to be down-regulated upon progression to invasive and malignant cancers (190–193). Epigenetic silencing of the BNIP3 promoter appears to be the most common mechanism explaining down-regulation of BNIP3 during malignant progression in lung, colorectal, hematologic, liver, and pancreatic cancers (194–200) although genomic deletion (201) and repression by specific microRNAs (202) has also been reported. Knockdown of BNIP3 in the 4T07 orthotopic mammary tumor model promoted tumor growth and metastasis (203) while genetic targeting of BNip3 accelerated the growth and metastasis of mammary tumors in the MMTV-PyVT mouse model of breast cancer (Chourasia et al., under review), both results supporting a tumor suppressor/metastasis suppressor function for BNIP3. Thus, similar to effects of Parkin deletion, loss of BNIP3 appears to promote tumorigenesis in mouse models consistent with a tumor suppressor function for mitophagy.

### Additional links between mitochondria and autophagy

Mitochondria contribute OMM lipids to nascent autophagosomes (204) and autophagosomes form at the junction of mitochondria with the ER (205). Given that ER associated with mitochondria also regulates sites of mitochondrial fission (60), we postulate that connections between the ER and the mitochondria may be important in coordinating mitochondrial fragmentation with autophagosome formation during mitophagy. Both BNIP3 and NIX have been reported to localize to the ER as well as the mitochondria (165, 206) but whether they are involved in such coordination is not yet clear.

Inhibition of autophagy can to lead to apoptosis in part due to accumulation of defective mitochondria that release cytochrome *c* and activate the apoptosome (207). Autophagy and apoptosis are coordinately regulated in part through modulation of Beclin-1 activity by Bcl-2/Bcl-X_L_ at the ER with Bcl-2/Bcl-X_L_ inhibiting autophagy by binding directly to Beclin-1 (208, 209). Conversely, autophagy proteins have also been shown to function in apoptosis with Atg12 acting as a BH3-only protein to inhibit anti-apoptotic Bcl-2 (210) while calpain cleavage of Atg5 induces a truncated form of Atg5 that can bind to and inhibit Bcl-X_L_ (211). While cleaved Atg5 promotes cytochrome *c* release and apoptosis (211), full-length Atg7 binds to p53 to prevent p53-dependent cell cycle and cell death (212). Various signaling pathways modulate this balance between autophagy and apoptosis. For example, starvation – induced Jnk1 signaling phosphorylates Bcl-2 disrupting its interaction with Beclin-1 (209). Under conditions of oxidative stress, nuclear HMGB1 is released to interact with Beclin-1 displacing Bcl-2 to promote autophagy (213). Conversely, apoptosis is promoted at the expense of autophagy as a result of calpain-mediated cleavage of key autophagy regulators, Atg5 and Beclin-1 (211, 214) and caspase-3 cleavage of Beclin-1, Atg4D, and GABARAPL1 (215, 216).

### Unanswered questions about role of mitophagy in cellular metabolism and cancer

Currently it is not clear to what extent the two major known mechanisms regulating mitophagy in mammalian cells (PINK1/PARKIN and BNIP3/NIX) (Figure 5) are dependent on each other or function independently. Interestingly, the mitophagy defect observed in Nix null erythroblasts can be rescued by mitochondrial depolarization with CCCP (169) suggesting that different mitophagy mechanisms may be somewhat redundant and explaining the lack of more severe phenotypes in mice genetically deleted for Parkin, BNIP3, or NIX (154, 160, 217). Recent work also identified a novel mechanism by which hypoxia promotes mitophagy, through dephosphorylation of the FUNDC1 protein at the OMM (218). De-phosphorylated FUNDC1 interacted with LC3 through a conserved LIR motif in FUNDC1 (218). This indicates that additional mechanisms regulating mitophagy may yet be discovered and suggest the existence of multiple redundant pathways modulating mitochondrial turnover.

How much mitochondrial damage or dysfunction can be tolerated by cells, and for how long, without loss of viability, is an additional unknown. Nor is it clear how rapidly cells accumulate damaged mitochondria once mitophagy is inhibited. The kinetics of mitochondrial damage accumulation will likely vary from cell type to cell type and in proportion to how much oxidative or metabolic stress is imposed. Studies examining a specific defect in mitophagy in adult liver in BNip3 null mice indicated that accumulation of defective mitochondria increased over time explaining increasing defects in cellular metabolism and lipid metabolism as the mice aged (161). Initially, there was accumulation of lipid and defective mitochondria in young mice, but over time increased hepatocyte cell death was observed and the mice developed steatohepatitis (161). Mitochondrial defects due to inactivation of mtDNA polymerase activity have been linked previously to aging (219, 220) with mtDNA mutations early in development causing respiration defects particularly in aging neural and hematopoietic progenitor cells (221, 222). Some of the aging effects are likely due to accumulation of ROS-induced mtDNA mutations in line with the “free radical theory of aging” since mouse life span can be increased and age-related phenotypes can be ameliorated through over-expression of mitochondrial catalase (223, 224). However, it remains to be determined to what extent defective mitophagy affects aging and which specific tissues are more susceptible to aging due to defective mitophagy. Clearly, since cancer is a disease of old age, defective mitophagy may contribute to tumorigenesis in an age-dependent manner. However, this remains to be formally tested.

### Significance of mitophagy for cancer treatment?

The duality of macro-autophagy function in cancer (both pro- and anti-tumorigenic, likely as a function of tumor stage, driving oncogene, and/or tissue type) makes it clinically questionable to generically target the entire autophagy process. However, a more effective therapeutic response in terms of long-term cancer patient survival may be possible by specifically targeting mitophagy. Inhibition of mitophagy increases ROS production at the mitochondria that may promote cell killing for at least a subset of tumor cells. Because mitochondria in normal cells are less likely to be dysfunctional and therefore less sensitive to mitophagy inhibition, by inducing ROS indirectly, we may avoid potentially harmful effects of supra-physiological ROS levels on normal cells. Furthermore, inhibition of mitophagy may disrupt fatty acid oxidation and Krebs cycle at the mitochondria and preferentially disrupt tumor cell growth that is also more dependent on mitochondrial citrate production for lipid synthesis than are normal cells. The combined effect of increased ROS and reduced mitochondrial metabolism arising from inhibition of mitophagy may be synergistic and promote efficient tumor cell killing. An alternative approach may be to combine mitophagy inhibition with drugs that induce other forms of mitochondrial stress signaling, such as Metformin that inhibits respiratory chain complex I or with antibiotics, such as tetracycline/doxcycline that inhibit mitochondrial protein translation, thereby inducing a “mitonuclear” protein imbalance and a mitochondrial unfolded protein response (UPR^mt^) (147, 148) that might be predicted to depend on mitophagy to clear damaged/dysfunctional mitochondria.

## Retrograde Signaling from the Mitochondria to the Nucleus in Cancer

**Figure 6:**
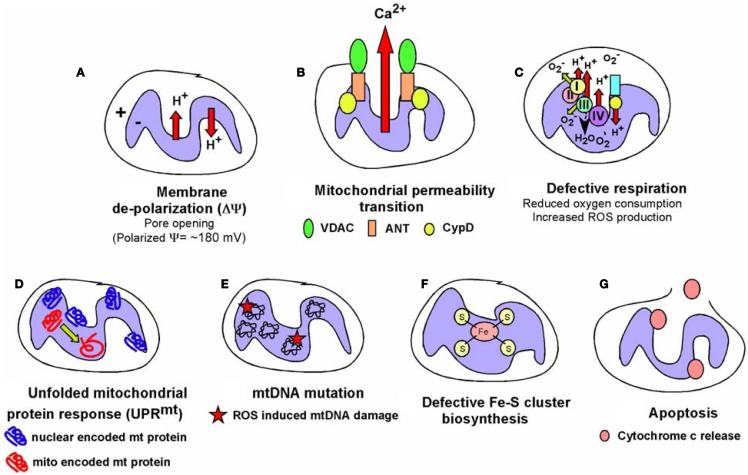
**Types of mitochondrial dysfunction**. We have attempted to define “mitochondrial dysfunction” in this review and the figure summarizes the major types of mitochondrial dysfunction that are known. **(A)** Mitochondrial inner membrane de-polarization (ΔΨ) during which there is loss of membrane potential; **(B)** mitochondrial membrane permeability transition (MPT) during which opening of the permeability transition pore (consisting of VDAC, ANT and usually association of Cyclophilin D) can lead to non-apoptotic cell death; **(C)** defective respiration/oxygen consumption due to altered expression of respiratory chain components, poisoning with respiratory complex inhibitors or many other stresses; **(D)** the Unfolded Mitochondrial Protein Response (UPR^mt^) can arise when there is an imbalance in expression of mitochondrial encoded mitochondrial proteins relative to nuclear encoded mitochondrial proteins, resulting in dysfunctional mitochondria; **(E)** damage to the mitochondrial genome most commonly reported as a result of oxidative damage to bases arising from respiratory chain defects; **(F)** defects in the production of iron-sulfur complexes in the mitochondrial matrix leading to defects not just in respiratory chain components but also other cellular enzymes; **(G)** release of cytochrome c anchored at the inner mitochondrial membrane via cardiolipin can result in formation of the apoptosome and activation of caspases leading to apoptosis. Some of these aberrant mitochondrial behaviors are inter-dependent, for example, membrane depolarization is a factor in mitochondrial permeability transition, defective respiration, and apoptosis amongst other consequences, but frequently can stand alone as a signal, for example to promote mitophagy. The consequences for the cell of these different forms of mitochondrial dysfunction are described in the text and below in Figure 7.

**Figure 7:**
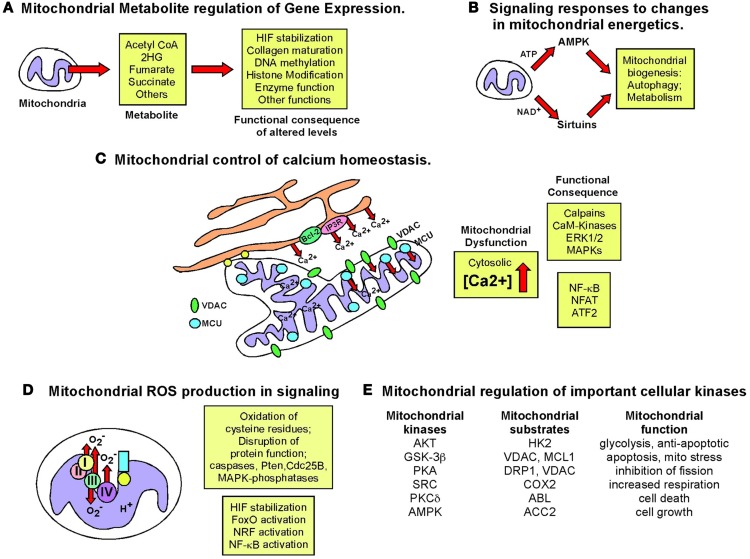
**Retrograde signaling from mitochondria to nucleus**. The role of the nucleus in regulating mitochondrial function has been examined extensively but the importance of mitochondrial events in signaling to the nucleus and to other cell growth mechanisms has been relatively under-studied. The figure summarizes some key signaling consequences of dysfunctional mitochondria. **(A)** Mitochondrial control of nuclear gene expression through effects of altered production of certain metabolites, such as α-ketoglutarate and succinate, on epigenetic modification of histones, stabilization of key transcription factors, such as HIF, in addition to effects on other enzymes and proteins. **(B)** Altered production of NAD^+^, ATP, and other changes in mitochondrial metabolism can modulate key signaling molecules in the cell, such as AMPK and the Sirtuins. **(C)** Mitochondria play a key role in buffering against Ca^2+^ flux into the cytosol from the extra-cellular environment or following release from the ER and failure of the mitochondria to execute this key function can result in altered Ca^2+^ signaling in the cell. **(D)** Mitochondrial ROS production has been one of the most extensively studied mediators of mitochondrial dysfunction and activity that elicits its effects on transcription factor activity as well as activity of key enzymes in the cell, such as caspases and phosphatases. **(E)** Important cellular kinases are known to localize to the mitochondria and altered mitochondrial dynamics and function may modulate the activity of these kinases not just at the mitochondria but at other sub-cellular locations if released from the mitochondria.

While most mitochondrial proteins are encoded by the nuclear genome and control of nuclear gene expression is key to mitochondrial function, it is also clear that mitochondria signal to the nucleus and such “retrograde” signaling is an area of increasingly important investigation (26). Mitochondrial dysfunction (Figure 6), defined as loss of membrane potential, defective respiration, defects in synthesis of iron-sulfur clusters, and/or the mitochondrial unfolded protein response (UPR^mt^), has been shown to alter nuclear gene expression through a variety of different mechanisms. For example, mitochondrial dysfunction can induce genome instability due to defective iron-sulfur complex synthesis in the mitochondrial matrix (225). Clearly, release of cytochrome *c* signals mitochondrial dysfunction and leads to apoptosis that is tumor suppressive, but short of inducing apoptosis, there are several other mechanisms of mitochondrial stress signaling that affect tumor cell growth. Altered metabolite levels, increased calcium (Ca^2+^) release from the mitochondria, elevated ROS production, reduced production of ATP or NADH arising from altered metabolism, changes in activity of mitochondrial kinases or other cellular enzymes/proteins dependent on redox state, Ca^2+^ levels or Fe/S complexes are among several major mechanisms put forward to explain how mitochondrial stress signaling affects nuclear gene expression (Figure 7), as discussed below.

### Mitochondrial control of epigenetics

Epigenetic control of nuclear gene expression is highly sensitive to mitochondrial function (226, 227). For example, levels of histone acetylation are regulated by availability of acetyl CoA produced from citrate exported from the mitochondria (228). In addition, fumarate, succinate, and other TCA cycle intermediates produced at the mitochondria regulate nuclear gene expression through effects on histone modifying enzymes, DNA demethylases, prolyl-hydroxylases, and other cellular dioxygenase enzymes (6, 13, 18, 226, 227, 229). Recent research progress in this area has been fueled by evidence showing that human cancer development is linked to mutations in genes encoding enzymes in the TCA cycle, such as isocitrate dehydrogenase-1 (IDH1) and -2 (IDH2) in glioblastoma and AML (230–232), as well as in fumarate hydratase (FH) and succinate dehydrogenase (SDH) in other rarer malignancies (6). Mutation of these genes in cancer leads to a buildup of their substrates; fumarate and succinate in the case of FH and SDH mutations (6, 226), or conversion of their regular substrate to a new “oncometabolite” in the case of mutant IDH1/IDH2 converting isocitrate to 2-hydroxyglutarate (229, 231). Inhibition of respiration through binding of the mitochondrial chaperone TRAP1 to SDH also resulted in elevated succinate levels and promoted tumor growth (233).

The key mechanism of action of these “oncometabolites” is their ability to compete with the structurally related α-ketoglutarate, a required co-factor for the afore-mentioned cellular dioxygenase enzymes (234–237) resulting in reduced activity of these key cellular enzymes. Amongst these enzymes are the prolyl-hydroxylases that promote turnover of HIF-α subunits, the TET2 DNA demethylase and the JmjC histone lysine demethylases (227, 229). Thus, the downstream consequences of increased fumarate, succinate, or 2HG include the accumulation of HIF-α and increased HIF target gene expression (235, 238), defects in collagen maturation (239), as well as hypermethylation and altered histone code due to disruption of epigenetic control of gene expression (236, 240, 241). This in turn was linked to altered gene expression patterns, defects in cellular differentiation and accumulation of immature stem cells and progenitors in affected tissues that can lead to cancer (240–244).

In addition to a role in inhibiting dioxygenases by competing with α-ketoglutarate for reversible binding, fumarate and succinate can also modulate cell growth by covalently modifying key signaling molecules. For example, elevated fumarate levels have been shown to stabilize HIF by covalently reacting with glutathione to produce an alternative substrate for glutathione reductase resulting in increased ROS production, as well as decreased NADPH (245). Succinylation of cysteine residues in KEAP1 in the cytosol results in activation of the NRF2 anti-oxidant pathway and up-regulated expression of stress response genes, such as heme oxygenase (246, 247). Similarly, succinylation of mitochondrial aconitase causes defects in iron-sulfur cluster binding (248). Tumor cells can tolerate such defects in the TCA cycle brought about by IDH1, FH or SDH mutation by redirecting the use of metabolites. In particular, glutamine can be used to generate citrate by reductive carboxylation (249, 250) or diverted to heme biosynthesis and degradation, thereby partially restoring NADH production and limiting accumulation of fumarate and succinate (251). This latter pathway is up-regulated in FH mutant tumor cells and inhibition of heme oxygenase specifically killed FH mutant tumor cells suggesting a novel therapeutic approach to treating cancers with FH mutation (251). Finally, the identification of novel protein modifications, including malonylation, succinylation, and butyrylation suggest the existence of novel regulatory pathways that may be sensitive to mitochondrial metabolites (252). Clearly, the more we understand about how metabolic pathways at the mitochondria are deregulated in cancer, the more likely it seems that we will identify novel signaling pathways that are aberrantly activated by accumulation or alternate fates of specific metabolites.

### Effects of mitochondrial dysfunction on calcium homeostasis

Mitochondria play a critical role in buffering intracellular calcium levels in part due to their localization close to calcium channels in the ER, such as the inositol-1,4,5-triphosphate-sensitive channels [Ins(1,4,5)P_3_R] that release Ca^2+^ from the ER in response to inositol-1,4,5-triphosphate (253). Mitochondria located at such microdomains of high Ca^2+^ concentrations rapidly take up the released divalent cation through the VDAC at the OMM and the mitochondrial calcium uniporter (MCU) at the IMM (253). While Ca^2+^-binding proteins can buffer cytosolic Ca^2+^ to some extent, the quantity of Ca^2+^ that the mitochondria can take up and buffer against is significantly larger. While VDACs are readily permeable to Ca^2+^ at the OMM and interact with Ins(1,4,5)P_3_Rs at the ER (254, 255), it has been suggested that levels and activity of VDAC may regulate the amount of Ca^2+^ that crosses the OMM (256). VDACs are subject to multiple levels of regulation including expression levels, post-translational modification, and protein–protein interactions all of which can limit Ca^2+^ uptake. Mitochondrial dysfunction results in increased cytosolic Ca2+, since only energized mitochondria can take up Ca^2+^ (253). Interestingly, members of the Bcl-2 family localized at the ER can modulate activity of the Ins(1,4,5)P_3_R and thereby regulate Ca^2+^ release and uptake by the mitochondria, with attendant effects on mitochondrial function and apoptosis (257).

The failure of mitochondria to take up Ca^2+^ effectively in response to its release from the ER or influx through the plasma membrane, directly affects mitochondrial activity. For example, key mitochondrial enzymes, including several TCA cycle enzymes are Ca^2+^ modulated (254, 258). Also, changes in mitochondrial matrix volume induced by altered Ca^2+^ uptake impact the activity of the ETC (258) and altered cytosolic Ca^2+^ concentration affects mitochondrial localization in the cell (259). Calcium inhibits mitochondrial movement in the cell through regulation of Miro (mitochondrial Rho GTPase), a Ca^2+^ binding Ras-like small G protein at the OMM that controls the interaction and movement of mitochondria along microtubules (259).

Failure of mitochondria to take up Ca^2+^ also results in aberrant activation of cytosolic enzymes such as calpain proteases (260) and Ca^2+^/calmodulin-dependent kinases (261) that can in turn alter cellular signaling cascades with dramatic effects on cell growth and viability leading to cancer (262). For example, mitochondrial stress induced in cultured cells through depletion of mtDNA (following growth in ethidium bromide) resulted in loss of mitochondrial membrane potential and elevated cytosolic Ca^2+^ levels that in turn led to increased glycolysis, increased ERK1/ERK2 and PKC activity (these enzymes are Ca^2+^ dependent), and increased tumor cell invasion associated with increased expression of cathepsin L and TGF-β (263, 264). Significantly, activation of calcineurin protein phosphatase by increased cytosolic Ca^2+^ in this system lead to dephosphorylation of IκBβ, and activation of NF-κB, as well as ATF2 and NFAT (265, 266). The pro-tumorigenic activities of NF-κB are well documented and include promoting resistance to apoptosis in addition to effects on cell migration and cell metabolism, through effects on HIF-1α (267–269). In summary, deregulated calcium homeostasis is one of the major consequences for the cell of dysfunctional mitochondria (Figure 7) that can result in dramatic changes in gene expression.

### Mitochondrial reactive oxygen signaling modulates cell growth and differentiation

As already mentioned above, mitochondria are the major source of cellular ROS and the contribution of ROS to mitochondrial stress signaling in cell growth, cellular senescence, and differentiation is significant (27, 29, 78, 270, 271). For example, ROS is required for KRas driven tumorigenesis (272) and anti-oxidants that quench ROS are anti-tumorigenic in certain systems (273, 274).

There are numerous mechanisms by which mitochondrial ROS can alter cell signaling but one of the major consequences of increased ROS and altered cellular redox state is the oxidation of thiol groups in cysteine residues in relevant proteins (27, 275). For example, the cysteine at the active site of caspases is inhibited by ROS production (276) as is the cysteine at the active site of many cellular phosphatases, including the Pten tumor suppressor (277), the CDC25B oncogene (278), and MAPK phosphatases (279).

The other key mechanism by which mitochondrial ROS is known to modulate cell signaling is through stabilization of HIF-1 subunits (280–284), as a result of prolyl hydroxylase inhibition (29). Increased HIF levels feed back to modulate mitochondrial respiration through induction of target genes such pyruvate dehydrogenase kinase-1 (PDK1) that inhibits conversion of pyruvate to acetyl CoA to feed the Krebs cycle and provide reducing agents for OXPHOS (285, 286), as well as the LON protease that degrades the regular COX4-1 subunit, and induction of COX4-2, an alternative isoform of COX4, that allows more efficient oxygen utilization and respiration under limiting oxygen conditions (287). NDUFA4L2, an inhibitory subunit of ND/complex I, is also induced by HIF-1α and limits respiration and ROS production (288). HIF-1α also protects cells from apoptosis associated with increased ROS, for example through induction of molecules such as ATIA that maintains mitochondrial thioredoxin 2 in a reduced form required for its anti-oxidant activity (289). Significantly, ATIA is up-regulated in human glioblastoma (289). Mitochondrial ROS-induced stabilization of HIF-α also explains in large part the pro-tumorigenic effect of deleting key regulatory molecules, including SirT3 (118), REDD1 (290), and BNIP3 (Chourasia et al., under review). Stabilization of HIF either through effects on accumulation of TCA cycle intermediates, as discussed above, or due to elevated mitochondrial ROS production, leads to increased angiogenesis, EMT, a switch to glycolytic metabolism, and priming of the pre-metastatic niche amongst many of the known tumorigenic effects of HIF activity (291–296).

FoxO transcription factors are another key signaling component in the response to elevated ROS levels and their induction activates not only anti-oxidant responses (increased expression of catalase and SOD2) but also cell cycle arrest and/or cell death (297, 298). For example, mitochondrial ROS in *Drosophila* as a result of inhibition of mitochondrial respiration lead to activation of a G1 cell cycle arrest in part due to activation of FoxO transcription factors (299). Interestingly, by antagonizing c-Myc, FoxO3a has also been shown to limit nuclear-encoded mitochondrial gene expression thereby limiting mitochondrial biogenesis under conditions of oxidative stress (300–302).

Finally, the KEAP1-NRF2 anti-oxidant signaling axis is activated by increased ROS due to the redox sensitivity of KEAP1 (303, 304). KEAP1 normally binds to NRF2 in the cytosol and promotes its degradation at the proteasome. ROS-induced dissociation of KEAP1 stabilizes NRF2 allowing it to translocate to the nucleus where it induces genes involved in quenching ROS (303). NRF2 is also stabilized by accumulation of p62/Sqstm1 that is often linked to defects in autophagy (305, 306). NRF2 stabilization promotes metabolic reprograming toward anabolic pathways, such as nucleotide biosynthesis thereby promoting tumor cell growth (307). NRF2 also promotes tumor cell survival by limiting levels of damaging ROS and constitutive activation of the KEAP1-NRF2 pathway has been detected in human cancers, either through activating mutations in NRF2 or through inactivating mutations in KEAP1 (308–310) and activation of NRF2 is associated with poor prognosis and therapy resistance (311). As a key regulator of mitochondrial biogenesis, as well as responses to ROS and autophagy defects, NRF2 activity is thus intimately linked to determining how the cell responds to mitochondrial dysfunction in terms of cell growth and tumorigenesis.

It is important to consider however that ROS has a relatively short diffusion distance in solution and thus mitochondrial ROS signaling may rely on proximity of ROS-producing mitochondria to their sites of action/targets. Intriguingly, perinuclear clustering of mitochondria induced by hypoxia was associated with increased nuclear ROS and was required for maximal HIF-1α DNA binding and target gene (VEGF) expression (41). These observations suggest that mitochondrial movement may play a role in allowing mitochondrial ROS to signal more directly to the nucleus. Perinuclear mitochondrial hubs that form in response to hypoxia and ROS (41) may also act to limit mitochondrial uptake of Ca^2+^ from the ER or the plasma membrane, thereby spatially regulating the effects of Ca^2+^ signaling in the cell.

### Altered mitochondrial metabolism signaling via AMPK and sirtuins

Defective oxidative metabolism and reduced ATP levels in cells activate AMPK (312) and certain drugs are known to induce AMPK as a result of inhibiting mitochondrial respiration, such as Metformin that inhibits complex I of the ETC and resveratrol that inhibits the F_0_F_1_ ATPase (313). AMPK plays a key role in mitochondrial homeostasis and while activated by mitochondrial dysfunction, feeds back to promote both mitochondrial biogenesis through activation of PGC-1α (314, 315) and mitophagy by activating ULK1 and inhibiting mTOR (316, 317), thereby improving the overall “health” of the mitochondrial pool in cells.

In addition to AMPK, the sirtuins serve as metabolic sensors of mitochondrial well-being due to their function as NAD+ dependent deacetylases (318). In particular, the mitochondrial sirtuins (SirT3, SirT4, and SirT5) are sensitive to the mitochondrial pool of NAD+ that is in turn determined by metabolic activity at the mitochondrion, with NAD+ levels increased by OXPHOS and reduced by fatty acid oxidation. The best characterized mitochondrial sirtuin, SirT3 deacetylates a number of critical enzymes involved in fatty acid metabolism (LCAD), the TCA cycle (IDH2), and OXPHOS (SDHB, complex I, II, V) in addition to cyclophilin D and UCP2 that modulate mitochondrial permeability and electron flow respectively (318, 319). The cytosolic and mitochondrial pools of NAD+/NADH are separate but can equilibrate through transfer via the malate-aspartate shuttle and thus mitochondrial metabolism may also influence nuclear and cytosolic sirtuins. Interestingly, nuclear SirT1 promotes mitochondrial biogenesis in response to nutrient deprivation through deacetylation and activation of PGC-1α (320), as discussed above, and similar to AMPK, SirtT1 may also promote mitophagy in response to nutrient deprivation through deacetylation of key autophagy genes, including Atg5, Atg7, and Atg8 (321).

### Mitochondrial localization of kinases involved in stress response signaling

Kinases known to play key roles in cellular stress responses have been detected at the mitochondria, including AKT, GSK-3β, PKA, ABL, PKC, AMPK, SRC, ATM, and others (313, 322–328). While substrates for some of these kinases at the mitochondria have been identified, the significance of localization of some of the other kinases is less clear.

AKT is a major growth promoting kinase that acts by inhibiting apoptosis in the presence of glucose and by activating mTOR (329). AKT also promotes glycolysis by phosphorylating hexokinase II (HKII) and promoting its interaction with VDAC at the mitochondria (328). HKII is required for tumor initiation and maintenance in mouse models (330). Failure of HKII to interact at the mitochondria with VDAC results in apoptosis (328), and thus, AKT plays a role in coupling mitochondrial metabolism with cell viability.

AKT also phosphorylates and inactivates GSK-3β a cellular kinase that localizes to the mitochondria under certain circumstances. Mitochondrial GSK-3β phosphorylates MCL-1 and VDAC amongst other mitochondrial targets (325, 331, 332). GSK-3β mediated phosphorylation of MCL-1 promoted its degradation and increased apoptosis (331), while phosphorylation of VDAC by GSK-3β resulted in increased mitochondrial membrane permeability, again predisposing to apoptosis (325, 333). Interestingly, GSK-3β also phosphorylates Drp1 resulting in elongated mitochondrial morphology that mitigates against cell death (334). This suggests that GSK-3β activity (either pro- or anti-apoptotic) is modulated by mitochondrial stress, although the precise mechanism of such a regulatory switch at the mitochondria is unclear. Obviously, GSK-3β is also known to phosphorylate and promote the proteasomal degradation of c-Myc, cyclin D1, and β-catenin (335–338) and thus one may postulate that activities of GSK-3β at the mitochondria influence nuclear oncogene activity. This would represent a novel perspective on retrograde signaling from the mitochondria to the nucleus.

Other kinases located at the mitochondria include PKA that associates with the mitochondria via adaptor molecules such as Rab32 and other A-kinase AKAPs (322, 339, 340) where it has been shown to phosphorylate VDAC (323), Drp1 (72), and other mitochondrial proteins. Localization of PKA to the mitochondria via AKAPs is subject to regulation by hypoxia and other physiological stresses (72, 341). For example, hypoxia destabilizes AKAP121 through induction of SIAH2, a mitochondrial ubiquitin ligase, thereby limiting oxidative capacity under conditions of low oxygen. Interestingly, AKAP121 also appears to promote mitochondrial localization of SRC-tyrosine kinase (342) where SRC appears to regulate CO activity and respiratory activity (342, 343), and other mitochondrial substrates for SRC family kinases are likely (344).

Association of protein kinase C-delta (PKCδ) with the mitochondria is induced by increased ROS (327) and this is turn recruits other signaling molecules, including the ABL tyrosine kinase that is associated with loss of membrane potential and non-apoptotic cell death (326). Again, whether these important kinases also play a role in mitochondrial function and signaling independent of cell death is not clear.

The localization of AMPK to the mitochondria is likely linked to its ability to modulate mitochondrial metabolism. Acetyl CoA carboxylase-2 (ACC2) is a well-established AMPK target that localizes to the OMM where it regulates lipid metabolism by controlling production of malonyl CoA (313). Inhibition of ACC2 (and ACC1) by AMPK boosts NADPH homeostasis under energy crisis and promotes tumor cell survival, anchorage independent growth, and tumor formation *in vivo* (345).

Finally, mitochondrial uncoupling activates ATM kinase, a fraction of which was shown to be located at the mitochondria (346). Loss of Atm in genetically engineered mouse models led to mitochondrial dysfunction suggesting the presence of a feedback loop, although the key substrates of ATM kinase in modulating mitochondrial homeostasis are not known (346). Of note, it has been reported that p53 tumor suppressor expression is sensitive to inhibition of mitochondrial respiration by unknown mechanisms (347) but whether p53 is the key substrate of mitochondrial ATM in mitochondrial stress signaling has not been examined.

In summary, there are numerous ways in which the mitochondria signals to the nucleus (Figure 7) and the consequences of mitochondrial dysfunction can therefore impact cell growth significantly.

## Oncogenic Control of Mitochondrial Function

A growing number of tumor suppressor genes and oncogenes are being investigated for their ability to regulate mitochondrial function either through effects on the expression and/or activity of components of the ETC or other key metabolic enzymes at the mitochondria or through effects on mitochondrial biogenesis and mitophagy. Some more recent findings in this area of seminal interest about two key tumor suppressors (p53 and RB) and two key oncogenes (Myc and KRas) and how they modulate mitochondrial function and metabolism are discussed here.

### The p53 tumor suppressor regulates mitochondrial function at multiple levels

The p53 tumor suppressor gene is the most commonly mutated gene in human cancer with inactivating mutations found in its DNA binding domain that result in loss of its normal transcriptional properties with gain of dominant negative or novel functions frequently the result (348, 349). Many of the tumor suppressor functions of p53 are attributed to its role as a transcriptional regulator of nuclear-encoded genes in response to stresses such as DNA damage, nutrient deprivation, and aberrant oncogene activity (350). The outcome of activating normal p53 in response to these stresses is induction of downstream target genes, such as p21^Waf1^ that induces a G1 cell cycle arrest or induction of pro-apoptotic genes like Puma and Bax (350, 351). While p21^Waf1^ is key to the ability of p53 to induce growth arrest (352), there are numerous downstream effectors of p53-induced apoptosis (350), including p53 itself (353, 354).

**Figure 8:**
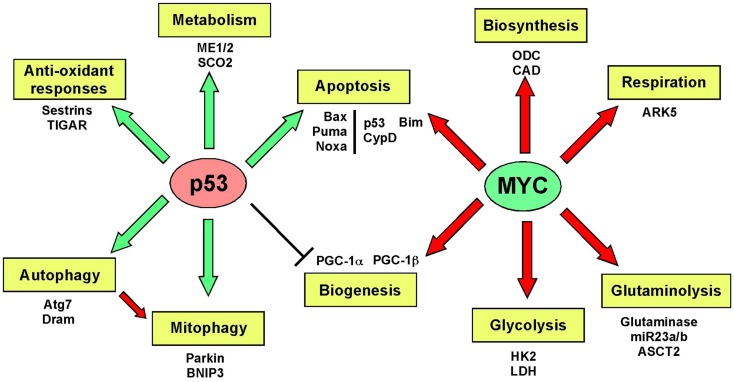
**Oncogene and tumor suppressor gene regulation of mitochondria**. The key activities of major oncogenes and tumor suppressors are increasingly being linked to effects on mitochondrial function. In particular, both p53 and Myc, inarguably two of the most significant tumor related genes in the human genome, have been shown to modulate several different aspects of mitochondrial function and in some instances, this has been shown to be key to their role in cancer, as discussed in greater length in the text.

In recent years, the ability of p53 to regulate cell growth processes, other than proliferation or apoptosis, has emerged, including roles for p53 in modulating expression of genes involved in mitochondrial biogenesis (through repression of PGC-1α), autophagy (355, 356), and mitochondrial metabolism (356, 357) (Figure 8). P53 indirectly affects mitophagy and mitochondrial quality control through induction of genes that regulate autophagy, such as Dram and Atg7 (355, 356). Recent work has identified a role for p53 in limiting the accumulation of damaged mitochondria in cancer by enforcing a growth arrest. Specifically, loss of Atg7 in KRas driven lung cancers caused accumulation of damaged mitochondria, defective fatty acid oxidation, and a growth arrest that retarded tumor growth (157). Inactivation of p53 alleviated growth arrest to some extent, although autophagy deficient tumor cells were unable to mobilize lipid stores and tumors continued to grow more slowly than control tumors that were functional for autophagy (157). Nevertheless, these results are consistent with a role for p53 in sensing defects in autophagy and/or mitochondrial function. Indeed, p53 may act more directly to modulate autophagy and responses to defects in autophagy (358) by interacting with Atg7 (212).

p53 also regulates expression of genes encoding regulators of the ETC [such as CO/Sco2 (359)], TCA cycle enzymes [such as malic enzymes ME1/ME2 (360)] in addition to modulators of glucose metabolism [such as HKII and specific glucose transporters (357)], and the pentose phosphate pathway [such as TIGAR (361)] in the cytosol. This transcriptional activity of p53 in metabolism has major significance for cell growth. For example, suppression of malic enzymes by p53 results in markedly lower NADPH production required for lipid synthesis and glutaminolysis (360).

p53 has been reported to be induced by AMPK through phosphorylation on serine 15 in response to nutrient stress resulting in a starvation-induced growth arrest (362) although it has been pointed out that the AMPK phosphorylation site in p53 (serine 15) is a weak AMPK consensus site (313) and thus perhaps that effects of AMPK activation on p53 are indirect. Given that serine 15 phosphorylation modulates the interaction of p53 with MDM2 to promote p53 degradation, reduced activity of AKT under nutrient deprivation resulting in lower nuclear MDM2 levels [AKT promotes nuclear localization of MDM2 and inhibits its interaction with p19/ARF (363)] may be a more likely explanation for increased levels of p53 in response to starvation. Nevertheless, p53 is induced by nutrient deprivation and its activity limits glycolysis and promotes OXPHOS, and indeed p53 null mice exhibit deficiencies in respiration and exercise performance (359).

In addition to these important functions as a transcriptional activator of genes that modulate mitochondrial turnover and metabolism at the mitochondria, p53 also plays a direct non-transcriptional role at the mitochondria. P53 has been detected at the mitochondria itself where it has been reported to promote cytochrome *c* release and apoptosis (353, 354). Specifically, mitochondrial p53 can function as a BH3-only protein that interacts with anti-apoptotic Bcl-2 and Bcl-X_L_ potentiating the pro-apoptotic activity of Puma, a target of nuclear p53 (364). More recently, mitochondrial p53 has been shown under conditions of oxidative stress to interact directly with cyclophilin D in the mitochondrial matrix. P53 uptake by mitochondria was dependent on mitochondrial membrane potential and interaction with mitochondrial chaperones (365). This resulted in opening of the mitochondrial permeability transition pore and induction of necrosis (365) that was not mitigated by nuclear functions of p53 in anti-oxidant gene expression induction (sestrins, glutathione peroxidase) but was blocked by cyclosporine-A, an inhibitor of cyclophilin D. Thus, p53 functions not just as a “guardian of the genome,” but also as a *guardian of mitochondrial integrity and function*.

### Modulation of mitochondrial metabolism and cell viability by the RB tumor suppressor

The RB tumor suppressor gene is commonly deleted in human retinoblastoma, osteosarcoma, and small cell lung carcinoma, but other genes upstream in the RB pathway, including p16/INK4A (CDKN2A) and Cyclin D1 (CCND1) are more commonly deregulated in human cancer than the RB tumor suppressor gene itself (366, 367). Indeed some cancers maintain functional RB at late stages in disease progression (368, 369) and it is not yet clear what the selective pressures are to maintain functional pRB at late stages of some tumor types but not others.

The RB tumor suppressor is considered primarily as a regulator of cell cycle checkpoints and induces a G1 arrest through repression of E2F transcription factors in response to numerous stresses, including growth factor deprivation, DNA damage, and hypoxia (370, 371). The role of pRB in establishing a cell cycle arrest has also been key to our understanding of how pRB promotes both cellular senescence (372, 373) and terminal differentiation (374–379). pRB/E2F complexes have also been shown to regulate genes involved in programed cell death, such as caspase-3, p73, and Apaf1 (380).

In addition to regulating genes involved in cell cycle control and cell death however, there has been over the past decade a growing appreciation that pRB and E2Fs together can modulate genes involved in metabolism and mitochondrial homeostasis (381), including BNIP3 (173), pyruvate dehydrogenase kinase 4 (382), as well as other mitochondrial genes (383). Recently, a role for RB in inhibiting glutaminolysis has emerged from studies in mammalian cells (83) and from metabolomic analyses in *Drosophila* (82). pRB was shown to repress expression of the glutamine transporter, and glutaminase-1 (83) and loss of RBF1 in flies led to metabolic reprograming such that glutamine flux to glutathione was increased and RBF1 deficient flies were more sensitive to oxidative stress (82). Thus RB/E2Fs are now considered to be significant modulators of cellular metabolism, although the relevance of these functions to the role of RB as a tumor suppressor *in vivo* remains to be determined.

Given the canonical role of RB/E2Fs as transcriptional regulators, it is not surprising that until recently attention was focused on effects of RB loss on expression of E2F regulated metabolism and mitochondrial genes (383, 384). However, like p53, pRB has also now been detected at the mitochondria (385, 386). pRB was shown to interact with and conformationally activate Bax to induce apoptosis (386), a function consistent with its role as a tumor suppressor. However, it remains to be seen whether other functions for pRB at the mitochondria can be determined that may explain the unexplained dependence on functional pRB at late stages of certain cancers (368, 369).

### Over-expression of c-MYC in cancer promotes dependence on functional mitochondria

The c-Myc oncogene is over-expressed in over 70% of all human cancers where it functions as a transcriptional regulator of genes involved in cell cycle (p21^Waf1^, cdc25A, Cdk4, cyclin D2), cell death (Bim, p53), replicative senescence (Tert, Bmi1) (387), genome stability [BRCA1/2, MUTS (388, 389), protein translation (ribosomal RNAs) (390)], cell adhesion (collagen, fibronectin, integrins) (391), angiogenesis (thrombospondin) (392), the tumor microenvironment (393), mitochondrial biogenesis (PGC-1β) (115), mitochondrial function (VDAC), and metabolism (glutaminase, lactate dehydrogenase-A/LDHA) (81, 394).

Myc-driven tumors regress rapidly when Myc is inhibited/turned off, as demonstrated in elegant switchable mouse models (395) and it has also been recently reported that KRas driven lung tumors are also dependent on Myc activity (396), highlighting the importance of c-Myc as a driving oncogene (81) and emphasizing the importance of how tumors become “addicted” to Myc. Induction of glutaminolysis at the mitochondria is a key factor explaining how tumors become “addicted” to Myc (80). Myc induces expression of key enzymes in glutaminolysis such as glutaminase (through repression of miR23a/b) (397) and ACST2 (the glutamine transporter) (80). Thus, Myc expressing tumors are dependent on glutamine as an anapleurotic source of carbon for the TCA cycle, as a source of nitrogen for nucleotide biosynthesis, to produce ATP and to generate lipids via reductive carboxylation at the mitochondria (249, 250). Withdrawal of glutamine causes Myc-driven tumor cells to apoptose (398, 399) and this is now being exploited for therapeutic purposes, as recently reported for N-Myc-driven neuroblastomas (400). A synthetic lethal screen identified Myc regulated molecules required to support glutaminolysis in c-Myc-driven tumors (401). Specifically, Myc was shown to induce expression of AMPK-related kinase 5 (ARK5) thereby promoting increased mitochondrial respiratory chain capacity required for glutaminolysis. Significantly, inhibition of ARK5 led to apoptosis of Myc-driven tumor cells (401), again with important therapeutic implications.

In addition to glutaminolysis, Myc regulates glucose metabolism by inducing expression of key glycolytic enzymes, including LDHA, HKII. Glycolysis is important in tumors not just as a low level source of ATP but also to provide precursors for biosynthetic pathways, including serine and nucleotide biosynthesis, and Myc promotes biosynthetic processes through induction of carbamoyl phosphate synthase, aspartate transcarbamylase, dihydroorotase (CAD), and ornithine decarboxylase (ODC) among other genes (81). Like Myc, HIF-1α also promotes glycolysis by inducing expression of glycolytic enzymes and when Myc expression is deregulated in cancers due to translocation or amplification, Myc, and HIF-1α cooperate to regulate glucose metabolism. However, hypoxia induces a growth arrest in normal cells and when Myc is expressed at normal levels in cells (not amplified or translocated), HIF-1α antagonizes Myc by displacing it from complexes with Max, by inducing Mxi-1, a repressive binding partner of Myc and by promoting Myc protein degradation at the proteasome (115, 296, 402). In this way, HIF-1α uncouples glycolysis from biosynthesis under hypoxic conditions and promotes mitophagy at the expense of biogenesis. By contrast, HIF-2α synergizes with Myc to stabilize Myc-Max dimers and to promote Myc target gene expression, cell growth, and genome stability (296, 388), although HIF-2α expression is more tissue restricted.

In summary, by increasing mitochondrial mass through induction of mitochondrial biogenesis (117) and promoting glutaminolysis at the mitochondria (80) (Figure 8), Myc oncogenes make tumors more dependent on mitochondrial function, not less. This may suggest that Myc-dependent tumors would be more susceptible to defects in mitophagy, mitochondrial fusion, or other key processes required for mitochondrial quality control.

### Activation of KRas

Activated KRas is one of the most prevalent oncogenic events in cancer of the pancreas, lung, and small intestine (403). In pancreatic cancer, activated KRas is linked to reprograming of tumor metabolism both through increased glycolytic flux to lactate, hexosamine biosynthesis, and non-oxidative pentose phosphate pathway (404). Also, KRas induces up-regulation of an alternative glutaminolysis pathway that converts glutamine-derived aspartate to oxaloacetate in the cytosol allowing pancreatic tumor cells to buffer against ROS through increased glutathione production (405). These results indicate that KRas driven tumors have evolved to be independent of mitochondrial metabolism (since both glycolysis and the alternative use of glutamine take place in the cytosol). However, other work points to a critical role for autophagy in KRas driven tumorigenesis by promoting mitochondrial metabolism (156, 157) suggesting that mitochondrial function is required for KRas tumorigenesis. It was suggested that KRas activation causes mitochondrial dysfunction, including increased ROS and reduced OXPHOS (406) but these studies were performed using a doxycycline inducible system that by itself induces mitochondrial dysfunction due to inhibition of mitochondrial protein synthesis and the UPR^mt^. Cells expressing activated KRas do exhibit reduced respiration associated with decreased expression of components of complex I of the respiratory chain (407) and activation of KRas does lead to increased c-Myc protein stability (408) suggesting that some alterations in mitochondrial function associated with KRas activation are in fact driven by increased levels of c-Myc.

## Conclusion

As the major energy and metabolite source in the cell, it stands to reason that mitochondrial function is deregulated in cancer and there is growing interest in understanding how altered mitochondrial function may be targeted to inhibit tumor growth. Emerging data identifies key oncogenes and tumor suppressors as modulators of different aspects of mitochondrial metabolism and dynamics. Interestingly, different tumor types may be more or less sensitive to modulation of mitochondrial function depending on which oncogenic lesions drive that tumor type. This is a new and exciting avenue in the continued “war on cancer.”

## Conflict of Interest Statement

The authors declare that the research was conducted in the absence of any commercial or financial relationships that could be construed as a potential conflict of interest.
